# Preclinical Cancer Models and Biomarkers for Drug Development: New Technologies and Emerging Tools

**DOI:** 10.4172/2155-9929.1000356

**Published:** 2017-06-30

**Authors:** Muthu Dhandapani, Aaron Goldman

**Affiliations:** 1 Integrative Immuno-Oncology Center, Mitra Biotech Woburn, MA 01801, USA; 2 Department of Medicine, Harvard Medical School, Boston, MA 02115, USA; 3 Division of Engineering in Medicine, Department of Medicine, Brigham and Women’s Hospital, Boston, MA 02115, USA

**Keywords:** Tumor biology, Cancer, Microorganisms, 3D organoids, PDX *in vivo* models

## Abstract

**Background and purpose:**

Predicting the efficacy of anticancer therapy is the holy grail of drug development and treatment selection in the clinic. To achieve this goal, scientists require pre-clinical models that can reliably screen anticancer agents with robust clinical correlation. However, there is increasing challenge to develop models that can accurately capture the diversity of the tumor ecosystem, and therefore reliably predict how tumors respond or resistant to treatment. Indeed, tumors are made up of a heterogeneous landscape comprising malignant cells, normal and abnormal stroma, immune cells, and dynamic microenvironment containing chemokines, cytokines and growth factors. In this mini-review we present a focused, brief perspective on emerging preclinical models for anticancer therapy that attempt to address the challenge posed by tumor heterogeneity, highlighting biomarkers of response and resistance.

**Recent findings:**

Starting from 2-dimensional and 3-dimensional *in-vitro* models, we discuss how organoid co-cultures have led to accelerated efforts in anti-cancer drug screening, and advanced our fundamental understanding for mechanisms of action using high-throughput platforms that interrogate various biomarkers of ‘clinical’ efficacy. Then, mentioning the limitations that exist, we focus on *in-vivo* and human explant technologies and models, which build-in intrinsic tumor heterogeneity using the native microenvironment as a scaffold. Importantly, we will address how these models can be harnessed to understand cancer immunotherapy, an emerging therapeutic strategy that seeks to recalibrate the body’s own immune system to fight cancer.

**Conclusion:**

Over the past several decades, numerous model systems have emerged to address the exploding market of drug development for cancer. While all of the present models have contributed critical information about tumor biology, each one carries limitations. Harnessing pre-clinical models that incorporate cell heterogeneity is beginning to address some of the underlying challenges associated with predicting clinical efficacy of novel anticancer agents.

## Introduction

Over the past several decades there has been an explosion in anticancer drug discovery research, ranging from novel general cytotoxic agents that broadly attack malignant features (i.e. rapid proliferation), to development of more focused compounds such as kinase-targeted small molecules that directly attack addictive oncogenes [1]. Despite the aggressive nature of this discovery effort, and the thousands of compounds developed and in-development, only 5% of lead drug candidates end up advancing through the clinic [2]. Indeed, a major limitation to drug development and clinical success remains our ability to predict patient outcomes before reaching clinical trial. The best preclinical model would be relatively inexpensive, amenable to high-throughput screening, and most importantly, reflect human-tumor biology as closely as possible. Indeed, this latter challenge underpins a major hurdle in the development of successful preclinical models for cancer drug discovery.

The notion that cellular heterogeneity limits the therapeutic success of drugs dates back more than seven decades to the original observations of Luria and Delbrück in microorganisms, which were later adapted to tumor biology [3]. Indeed, more recent efforts in basic biology and clinical evidence have begun to uncover just how integral tumor heterogeneity is for therapy response and resistance. For example, the earlier discovery that small populations of inherently drug resistant cancer cells exhibiting stem-like features [4] has been overshadowed by newer evidences that stochastic gene expression [5] or non-genetic cell state dynamics arising from spontaneous phenotypic switching [6] are just the ‘tip of the iceberg’. Indeed, our own research has recently revealed that different cell states can even be induced by drug pressure, itself [7,8] via deterministic mechanisms [9]. Such evidences beg the question: what are novel methods we should be employing to study the preclinical efficacy of drugs, which incorporates the inherent dynamic, stochastic and deterministic processes that underlie response and resistance?

Despite rigorous efforts to design novel platforms for drug discovery, preclinical cancer models have been challenged by their inability to faithfully map to patient outcomes [10–13]. While much of the early cancer drug discovery was performed using *in-vitro* conditions in cell-based models that poorly represent actual malignancies, here we will describe some emerging tools based on more complex co-culture technology using live cell *in-vitro* and human explant experiments, as well as discussing *in-vivo* platforms currently in use. As described below, we argue that preclinical models, which introduce inherent biological complexity, preserve the intrinsic dynamism of cellular heterogeneity, and maintain the 3-dimensional architecture of the native tumor, will lead to improved strategies for drug development.

## Present Tools or Models

*In-vitro* preclinical cancer models have been a mainstay of research since the first cancer cell line was established from humans [14]. In the past several decades, techniques and tools have been improved by moving from 2-dimensional cell culture, to more improved 3-dimensional cell growth, which better recapitulates the physiologic environment and growth patterns of solid tissue and tumors [15].

### From 2-D cell line models to patient derived 3-D organoids

Pre-clinical research to delineate molecular mechanisms that drive cancer growth and progression is usually carried out in 2-dimensional (2-D) cell culture systems, which are efficient and reliable, but lack the appropriate cell-cell contact environment typically observed *in vivo.* However, some successes using these less complex models have been noted. For example, ChemoFx - a 2-D culture based chemoresponse selection marker, has shown some clinical benefit and utility in gynecological cancer [16–18]. The ChemoFx^®^ Assay harnesses and platform (a phenotype-based, using a short-term culture) designed to predict the sensitivity and resistance of a given patient’s solid tumor to a variety of chemotherapy agents. A portion of a patient’s solid tumor, as small as a core biopsy, is mechanically disaggregated and established in primary culture where malignant epithelial cells migrate out of tumor explants to form a monolayer. Cultures are verified as epithelial and exposed to increasing doses of selected chemotherapeutic agents. The number of live cells remaining post-treatment is enumerated microscopically using automated cell-counting software. The resultant cell counts in treated wells are compared with those in untreated control wells to generate a dose-response curve for each chemotherapeutic agent tested on a given patient specimen. Features of each dose-response curve are used to score a tumor’s response to each *ex-vivo* treatment as “responsive,” “intermediate response,” or “non-responsive.” Collectively, these scores are used to assist an oncologist in making treatment decisions.

Despite using patient-derived cells and tissue, 3-dimensional architecture and preserved heterogeneity of tumor cells *in-vitro* is a more accurate model for the complex microenvironments and surrounding stromal components. These more complicated *in-vitro* models are termed ‘organoids’ [19]. Organoids are developed by explanting dissociated patient-derived cells into a semi-solid extracellular matrix and expanding these cells in growth-factor-enriched medium [20]. Organoids have the distinct advantage of growing in three dimensions, and they often recreate the endogenous architecture of the tissue from which they were derived, theoretically recapitulating the *in vivo* tumor environment more closely than 2D cultures on plastic, enabling maintenance of the same driver mutations that were identified in the primary tumor. Recently, organoids have been developed from patients with multiple cancer indications, each one requiring unique scaffolds and stromal components [20–22].

Excitingly, new 3D culture systems are beginning to incorporate advances in biomaterials, microfluids and tissue engineering to improve culture quality and reproducibility. Indeed, microfluidics can not only empower methods of isolation and downstream manipulation of circulating tumor cells (CTCs) from the blood of patients with cancer have dramatically improved over the past few years [23]. More importantly, these techniques enable researchers to capture the inherent shear fluid pressures that are found in the native microenvironment of the tumor, a feature that can ‘turn on’ inherent drug resistance mechanisms, and dynamically influence heterogeneity of spheroidal cell clusters [24].

### PDX *in vivo* models

While *in-vitro* models enable high-throughput screening of drugs, they fail to take into account the full complexity of a living organism. *In-vivo* models use patient biopsy material implanted subcutaneously or orthotopically and expanded *in vivo*, which have the theoretical advantage of retaining some of the histology, gene expression and somatic genetics of the patient tumor [25]. PDX models are becoming standard in the drug discovery pharmacology toolbox for testing efficacy; they have also been suggested as avenues for selecting patient therapies [26]. Recently, unique *ex-vivo* live tissue sensitivity assay (LTSA) based PDX model reflected clinical patients’ responses and this could be used as a personalized strategy for improving systemic therapy effectiveness in patients with pancreatic cancer [27]. However, this approach requires a significant amount of lead-time, redering acute treatment decision-making difficult.

Although animal models have been an exciting advance in our understanding of drug effect, pharmacodynamics and pharmacokinetics, they are limited by the inability for high-throughput screening given the expensive, time consuming and laborious efforts required. CIVIO, an *in vivo* based technology platform, enables simultaneous assessment of up to eight drugs or drug combinations within a single solid tumor [28]. The platform is currently designed for use in animal models of cancer and patients with superficial tumors but can be modified for investigation of deeper-seated malignancies. This CIVIO technology essentially allows for medium-throughput screening of drug activity in living animals and this application has been tested in human xenografted mouse models including a model of chemoresistant lymphoma, canine and in human patients [28].

### Explant, organotypic culture

Organotypic tumor slices retain the complexity of tumors *in vivo* without extensive manipulation of the tissue. In other words, they preserve the 3D environment of the native tumor or to preserve the heterogeneity of the original tumor admixed with stromal cells. Live thin sections of the original patient solid tumor maintained in commercial culture plate inserts have been treated with drugs; under these conditions, the tumor cells showed appropriate on-pathway responses to inhibitors Preclinical model of organotypic culture for pharmacodynamic profiling of human tumors. Using such improved methods recently cytotoxicity responses of individual tumor slices to chemotherapy was assessed in multiple solid tumors [29,30].

CANScript^™^ is a rapid reproducible *ex-vivo* tumor explant system, developed to mimic native tumor microenvironment. By including autologous paracrine growth factors, cancer specific customized matrix support, autologous immune environment along with other growth promoting conditions, significant improvements were observed in viability, proliferation of tumors including retention of tumor/stroma, cancer phenotypes, integrity at micro-architecture level and maintenance of functional signaling network. By recreating of the complete tumor microenvironment, CANscript^™^ evaluates how a unique patient’s tumor responds to tested treatments, in real-time. Unlike alternative platforms, CANscript^™^ does not manipulate or distort tumor tissue for evaluation [31].

## Biomarkers for Preclinical Modeling

### Screening apoptosis

While pre-clinical models for cancer pose their own fundamental biological challenges, screening drugs requires a concerted effort to identify specific biomarkers that infer anticancer efficacy. As a strategy to improve the biomarkers predictive of clinical response or resistance, novel platforms have been engineered. For example, ChemoINTEL (original name MICK Assay) measures *in-vitro* apoptotic response of a patient’s tumor to chemotherapy drugs using multiple biochemical and morphologic apoptotic markers within single cells continuously over a 48 hour cell culture period. Based on foundational work done at Vanderbilt University, ChemoINTEL is a new category of chemo sensitivity assay relying on drug-induced apoptotic response in cell lines rather than classic phenotypic markers that have been employed for decades [32].

### BH3 as an emerging target

In addition to apoptosis, cytotoxic chemotherapy targets elements common to all nucleated human cells, such as DNA and microtubules, which induce cell death in tumor cells through unique pathways. Clinical response to these drugs correlates with, and may be partially governed by, the pre-treatment proximity of tumor cell mitochondria to the apoptotic threshold, a property called mitochondrial priming. BH3 profiling is used to measure priming in tumor cells from patients with multiple myeloma, acute myelogenous and lymphoblastic leukemia, and ovarian cancer. This assay measures mitochondrial response to peptides derived from pro-apoptotic BH3 domains of proteins critical for death signaling to mitochondria. Patients with highly primed cancers exhibited superior clinical response to chemotherapy. In contrast, chemoresistant cancers and normal tissues were poorly primed. Manipulation of mitochondrial priming might enhance the efficacy of cytotoxic agents [33].

## Conclusion and Perspectives

**Figure 1:**
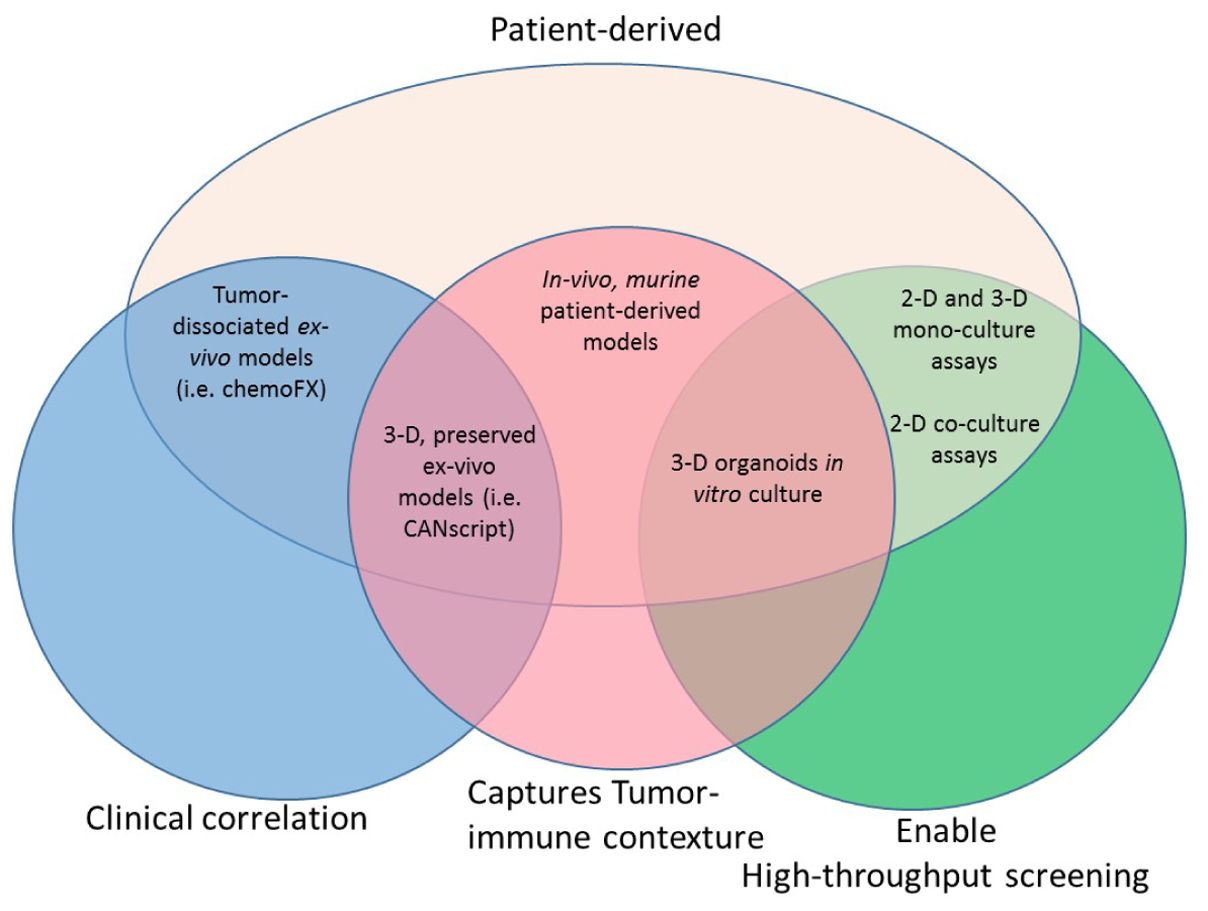
Clinical correlation using autologous systems.

Drug development is only going to accelerate in the next several decades. Indeed, drug combinations, ease of identifying new targets, and improved medicinal chemistry techniques are making drug development more simple and inexpensive than ever before. Moreover, we are entering a new era in medicine in which novel compounds are now being introduced to target, and re-awaken the body’s own immune defense to fight cancer. The renaissance in cancer immunotherapy is bringing with it added complexity for preclinical drug-development tools. While some models enable high-throughput screening of drugs, they cannot accurately re-capitulate the tumor-immune contexture, and complexity of the microenvironment. Other models that rely on animal systems, like *in-vivo* murine PDX models, fail to faithfully correlate to the clinical context given the interspecies dependence. Finally, some researchers have attained an elegant admixture of models, which can re-create the microenvironment, retain tumor-immune contexture and keep clinical correlation using autologous systems. This latest example, although robust, still requires further development to enable high-throughput screening, which will advance our capability and meet the demands of future drug development (Figure 1).
